# How to … Implement a Successful Medical Student–Staff Partnership Project

**DOI:** 10.1111/tct.70151

**Published:** 2025-07-13

**Authors:** Cate Goldwater Breheny, Eve O'Connell, Rasha Mezher‐Sikafi, Mike Streule

**Affiliations:** ^1^ Imperial College School of Medicine London UK; ^2^ School of Public Health, Imperial College London London UK; ^3^ Imperial StudentShapers, Education Office Imperial College London London UK

**Keywords:** curriculum, how‐to guide, medical education, student–staff partnership

## Abstract

Student–staff partnerships, where students work as equal partners alongside staff, are a powerful process to develop learning and teaching in higher education. However, within undergraduate medical education, there are multiple challenges that restrict medical students' and staff's ability to engage in partnership work. We draw on our experiences of a successful medical student–staff partnership in summer 2023 to identify four key barriers to successful partnerships: lack of time, emotions and hierarchy, lack of awareness of professional identity formation and ineffective use of student expertise. By naming and exposing these challenges and sharing our experiences and tips on how they can be overcome, we provide educators with a comprehensive guide to implementing successful student–staff partnership within the undergraduate medical education setting. We conclude that reflective practice in partnerships provides a valuable opportunity for learning and personal development for both staff and students.

## 1 Introduction

Student–staff partnerships in higher education, where students work as colleagues and co‐creators alongside staff, are increasingly valued [1]. However, to our knowledge, undergraduate medical student–staff partnerships remain relatively uncommon. This is due to many student and staff factors, from timetable restrictions for medical students to teachers balancing clinical commitments with educational work. Pronounced medical hierarchies mean staff may be less receptive to working with students [2], creating a cultural barrier to the transition to seeing each other as mutual partners required for student–staff partnership [3].

**FIGURE 1:**
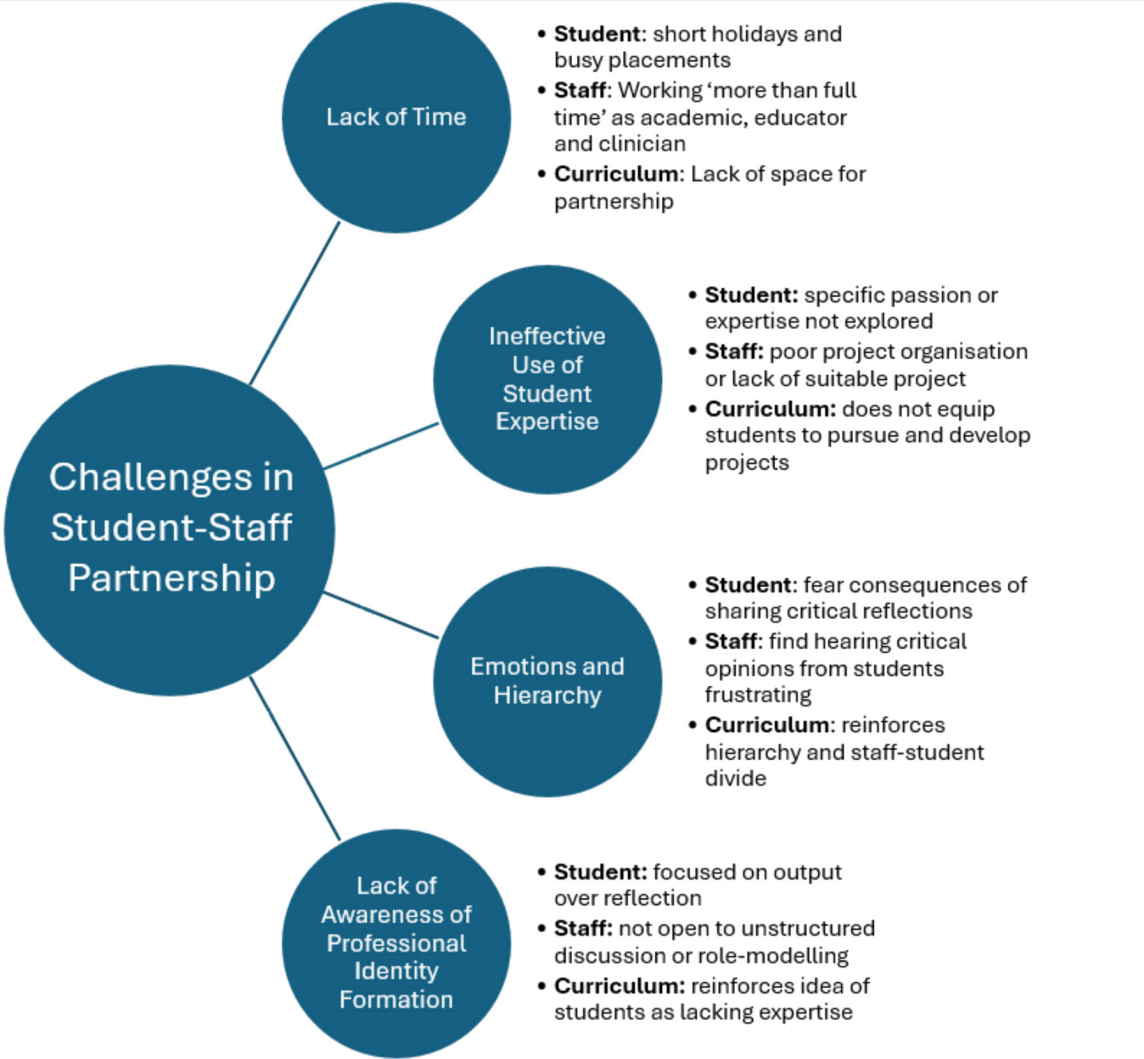
A conceptual diagram demonstrating the four key challenges of student‐staff partnerships and their impacts on students, staff and curriculum factors.

We reflect on our experience of a successful medical student–staff partnership and the literature to empower medical educators to benefit from and expand student–staff partnership. We identify four key challenges to student–staff partnership in relation to student, staff and curriculum factors (see Figure 1): lack of time, emotions and hierarchy, lack of awareness of professional identity formation and ineffective use of student expertise.

‘We identify four key challenges to student‐staff partnership in relation to student, staff and curriculum factors’.

## 2 Background

The authors undertook a student–staff partnership at Imperial College School of Medicine (ICSM) in summer 2023. This was a 5‐week paid project between the Professional Values and Behaviours (PVB) staff team and two year three medical students through Imperial's established StudentShapers scheme. The scheme is an established part of Imperial's Learning and Teaching Strategy, offering students paid opportunities to undertake partnership projects to improve curricula, develop innovative teaching practices and make positive change to the student experience [4]. PVB is a spiral domain of the ICSM medical course covering medical ethics and law, quality in healthcare and professional identity formation and behaviours. PVB aims to nurture students as they develop their professional identity and start to ‘think, act and feel’ like doctors [5].

The authors of this paper are the two student partners and the staff partner who undertook student partner recruitment and partnership throughout the project. Throughout this piece, ‘student partner’ refers to the students who engaged in the student–staff partnership process, and ‘staff partner’ refers to the faculty member who did the same. Where we have all reflected together, we use ‘we’ to refer to our shared opinion and values, and we write from a stance of hoping to encourage increased student‐staff partnership activity in medical education. Our final author was the lead on Imperial's StudentShapers scheme at the time of the project and shared his insight of student–staff partnership from a leadership perspective.

Over 5 weeks, student partners were involved in multiple elements of pedagogical development and curriculum design. Student partners worked flexibly across a 4‐day week aligned with staff partners. They were involved in a broad range of activities, such as the following:

- Feeding back on students' perceptions of a newly introduced professionalism‐focused practical exam station and a proposed student portfolio
- Reviewing and rewriting cases used to prompt discussion in small group teaching
- Redesign and delivery of a year 1 quality improvement session
- Developing and delivering a student‐led component of personal tutor training

The student partners' contribution led to them being recognised externally with a Student Partnership Award and their contribution becoming embedded across the years one to three PVB curriculum [6].

## 3 Challenge 1: Lack of Time

Students and staff can struggle to engage with student–staff partnerships due to lack of time [7, 8]: partnerships at ICSM therefore often take place in student partners' summer vacation time [4]. However, medical students, especially in the clinical years of their degree, have short breaks with limited time for rest or paid work to fund their studies. These breaks also represent opportunities for leave, clinical work and curriculum review for educators. Large amounts of limited project time can therefore be taken up with introductory and handover periods, impacting productivity and motivation. Our partnership further required longitudinal commitment beyond the project at hand to ensure changes were sustained and work was fully completed and implemented. This is challenging in medical partnerships, limiting their long‐term impact. Ultimately, clinical staff and medical students are more likely to find that projects have lower output or impact, making individuals less motivated to continue and invest the necessary time required for partnership approaches [1, 7].

The staff partner addressed this in two key ways. Firstly, they invited fellow educators to work with student partners on their available projects, reducing the commitment level for any single staff member and broadening collaboration. Secondly, a clear induction timetable was created prior to the commencement of the project to make the best use of the initial week and ensure induction meetings were scheduled in both student and staff calendars. Together, we also established the aims and vision of the overall project in the first week, including aligning expectations and coordinating regular touchpoint meetings throughout the 5 weeks. This dedicated time at the start of the project allowed student partners to feel a greater connection with the wider PVB team and began the process of developing strong student–staff relationships.

‘dedicated time at the start of the project allowed student partners to feel greater connection’.

## 4 Challenge 2: Emotions and Hierarchy

Students and teachers' different and sometimes conflicting curriculum experiences can act as a barrier to successful student–staff partnership [8]: partners often come to projects with divergent or even conflicting agendas [3]. In medicine, pre‐existing hierarchies [2] mean that staff can be predisposed to a particular mindset that diminishes students' contributions as uninformed or naive, while medical students may think that staff will not value their differing opinions. Student partners found the transition from seeing staff as our teachers to our colleagues challenging in this clinical culture [9], creating barriers to the strong rapport student–staff partnership requires [7, 10].

Reciprocal dialogue between staff and students requires an active commitment to renegotiate roles [8]. We adopted a proactive approach to try and break down these barriers across the relatively short 5‐week duration of our project—acknowledging that this change in roles was, to an extent, temporary. We began the project by sharing our own ambitions for change in the curriculum in unstructured brainstorming time. We committed to calling each other by first names throughout, which facilitated a transition to seeing each other as colleagues. Across the project, we had regular weekly touchpoint meetings where we could all debrief and share any concerns in a safe space. Building these in, rather than putting the onus on student partners to raise concerns across a power differential, allowed issues to be resolved quickly. At the end of the project, we went for lunch together, cementing a more equal relationship.

**BOX 1 A case study example from our project of how working to dismantle medical hierarchies led to positive educational outcomes.**

The staff partner identified placement debriefing sessions, which were historically poorly attended by students in their third year, as an opportunity for student partner input. To discuss why students did not engage with these sessions, honest discussions were needed. Student partners took time to feel safe exploring negative student views on the sessions. Student partners ultimately highlighted several problems, from timetabling to scaffolding of learning: students did not understand the role of these sessions or have time to travel to attend them. Student partners were then encouraged to think creatively about what could be improved, and faculty were encouraged to consider the realistic implementation of these ideas. The student partners' fresh perspectives brought a new excitement to the teaching, and we considered elements of design, timetabling and implementation from a student perspective. They encouraged faculty to change the structural timing of the sessions, including the development of a new asynchronous learning session that included a peer–peer voice. Multiple changes were made because of the candid discussions that took place. Without the passion and creativity of the student partners, it is unlikely that such positive changes would have been made. The implementation of the new process in the following academic year resulted in improved student attendance and engagement.

‘Reciprocal dialogue between staff and students requires an active commitment to renegotiate roles’.

## 5 Challenge 3: Lack of Awareness of Professional Identity Formation

Student–staff partnerships allow students to develop their identities as learners and people [1, 11]. However, there is, to our knowledge, no discussion of the potential interaction between student–staff partnership and healthcare students' developing professional identities. Cruess et al. [12] conceptualise medical professional identity as involving a process of socialisation through student clinical interactions. Student–staff partnership offers opportunities for medical students to work equally alongside clinical staff, thus developing their socialisation into a professional identity, but all partners may not be aware of this.

‘Student‐staff partnership offers opportunities for medical students to… develop their socialisation into a professional identity’.

Student partners found that staff partners acted as role models. If this is not discussed, a chance for medical students to benefit from professional development and mentoring relationships with staff could be missed. For example, student partners observed staff partners' delivery of constructive feedback and went on to develop their skills. Equally, staff partners discussing professional identity with student partners can appreciate issues that may otherwise remain obscure [13].

**BOX 2 A case study example from our project of how we discussed and learned around our experiences of professional identity development.**

We discussed how best to support students around career decisions during our StudentShapers project. Staff based on campus do not have full insight into the experiences students have on placement and how this impacts their views of their future career options, while students often do not realise this is a blind spot for staff and instead feel ignored in their career development. Through open, reflective conversations, student partners explored how, on busy placements, students rarely have the time to have conversations around careers with doctors. We decided that videos in conversation with specialty trainees would give students a snapshot of what a conversation about careers looks like as well as insight into that specialty. This culminated in us producing a series of videos with cardiothoracic surgery trainees. Student partners were able to reflect on what questions felt most relevant to us and discuss with peers interested in surgical careers to make the videos most useful to students. Also generating the videos, having honest discussions between student and staff partners about careers on placement prompted student partners to reflect on our professional development and connect with doctors at a different stage of their career and training, a rare opportunity in medical school.

## 6 Challenge 4: Ineffective Use of Student Expertise

Student–staff partnership requires consideration of what areas of teaching and learning will most benefit from this approach, [1, 7]. We found that the most useful areas for medical student input centred on student engagement and the hidden curriculum, the cultural influences and ideas transmitted and reinforced on clinical placement [14]. Students are in a unique position to provide input as a group who actively experience both the hidden and explicit curricula.

‘the most useful areas for medical student input centered on student engagement and the hidden curriculum’.

Student partners found their insight particularly useful in addressing issues related to the placement‐based learning environment where students learn about team interactions, experience positive and negative role modelling and enact hierarchies within their hospital‐based communities of practice [5, 13, 14]. Faculty based on campus often do not have complete insight into student experiences in placement‐based learning, leading to teaching that does not feel representative to students.

**BOX 3 A case study example from our project of how we used our experiences of the hidden curriculum on placement to develop a new session.**

The NHS industrial action and the general working conditions of junior doctors are understandably an area of significant concern and discussion both amongst medical students but also between other healthcare professional and medical students. Often when these discussions are undertaken on placement, central faculty are unaware that they are occurring. Student partners began the partnership keen to work on a project which discussed the industrial action undertaken by junior doctors and found that staff partners had similarly considered an ‘out of hours session’ to discuss such relevant topics. Student partners were then given the opportunity to work with staff in a way that could bring this sensitive area into a fruitful session. They were encouraged to draw on our experiences with other students and on placement to guide what the session could address and how it could be structured to stimulate productive debate and reflection. Particularly, they focused on the pressure to leave the NHS and attitudes around the strikes. The result was a new session which brought together several doctors with early years medical students to discuss the issues in a structured manner guided by a facilitator.

## 7 Conclusion

There is huge potential for medical student–staff partnerships within undergraduate medical education. We identify and explore the four main challenges we experienced for a guide to success in medical student–staff partnership. Student–staff partnership is a skill set that can be developed, learned and applied, focusing on open and honest reflection: Table 1 summarises our key tips. Reflection and practicing working together are important to develop your own expertise in student–staff partnership. Like any team, building a relationship between staff and student team members is a vital part of this skill set. Understanding student–staff partnership as skill‐based changes mistakes into opportunities for learning. There will be elements that work less well in the partnership: these are learning opportunities, not failures. Honest and meaningful student and staff reflection before, during and after the partnership enables learning and skill development beyond individual project outputs.

**TABLE 1:** A table overview of our practice points for success in medical student–staff partnership, outlining the key challenges we identified, the negative outcomes they lead to and how our practice points overcome these challenges.

Challenge	Negative outcomes if not addressed	Tips to overcome
*Lack of time*: Both medical students and staff often lack dedicated time for student–staff partnership	Poor engagement with work and lack of output Supervision meetings can be difficult to arrange: students feel unsupported Focus on superficial issues or outcomes as ‘no time’ to look deeper	Have clarity over the project length and the expected commitment in that time: Staff and students should have paid time specifically dedicated to the project Trust each other to work autonomously Frequent, scheduled touchpoints and deadlines help to keep student and staff on track
*Emotions and hierarchy*: The complex emotional dynamics in the medical hierarchy can impede open and authentic communication	Meaningful feedback is not given so solutions do not address real issues Breakdown of student–staff partnership due to mistrust or lack of rapport Avoidance of focus on problem areas within the curriculum so deeper issues are not addressed	Discuss how to give feedback in advance and set ground rules, such as not raising problems without solutions. Consider giving more critical feedback over email or in a document, giving the other person time to process and respond Make time and effort to build rapport and space separate from the hierarchical clinical setting, such as by using first names and agreeing mutual constructive aims for the project Scheduled, regular debriefing sessions allow emotions to be raised safely Ensure principles are followed equally by all staff and student partners
*Lack of awareness of professional identity formation*: This is a key opportunity for medical students to develop confidence and skills which may be missed	Students and staff lose an opportunity for a long‐term role modelling or mentoring relationship Professional and skill imbalances between partners are not addressed or discussed, hindering relationship formation and learning opportunities Staff do not gain feedback on student perceptions of the hidden curriculum and professionalism as the topic is seen as taboo	Student–staff partnerships offer students a chance to have a more meaningful relationship with clinical medical educators whom they often see as role models than is normally available in the curriculum: lean into discussions around medicine and careers! Recognise that staff partners act as professional role models to student partners Consider which students you will target and their stage in their professional journey: You may well get very different feedback from first versus final year students and students are best placed to act as partners on areas of the curriculum they have just experienced
*Ineffective use of student expertise*: Staff need to select suitable and useful projects where student partners can give helpful input	Student partner views can be dismissed by staff as not relevant or useful overall, rather than on a specific topic Students may feel disheartened if asked to work on a project they do not understand or do not feel is relevant to them and disengage	Planning for the partnership should take place in advance of the project starting, with decisions made around the best way to utilise student partners Exploring student interests at the start of the partnership helps keep students motivated and reveal the most useful projects

## Author Contributions

**Cate Goldwater Breheny:** conceptualization, writing – original draft, writing – review and editing. **Eve O'Connell:** conceptualization, writing – original draft, writing – review and editing. **Rasha Mezher‐Sikafi:** conceptualization, writing – original draft, writing – review and editing, supervision. **Mike Streule:** writing – review and editing.

## Conflicts of Interest

The authors declare no conflicts of interest.

## Data Availability

Data sharing not applicable to this article as no datasets were generated or analysed during the current study.
